# Identification of the chemical profile and evaluation of the antimicrobial effect of *Eryngium billardieri* Delar essential oil component against bacterial species of agricultural and food interest

**DOI:** 10.3389/fmicb.2023.1249780

**Published:** 2023-10-12

**Authors:** Habibeh Hajian-Maleki, Masoud Shams-bakhsh

**Affiliations:** Department of Plant Pathology, Faculty of Agriculture, Tarbiat Modares University, Tehran, Iran

**Keywords:** disk diffusion, GC–MS, antibacterial preservative, essential oil, Asia

## Abstract

Studies on the antibacterial activity of the essential oil of *E. billardieri* are limited. In this study, we identified this herb as a natural complex effective against several bacteria by employing disk diffusion and broth microdilution susceptibility methods. Primary estimation of the antimicrobial effect of this herbal compound by disk diffusion method showed that the oil could inhibit the growth of the tested bacteria by the appearance of haloes between 8.25 and 21.25 mm. In the next step, the oil was found to be active against all 24 tested Gram-negative and Gram-positive bacteria in the broth media, at minimum inhibitory concentrations ranging from 0.67 to 34.17 g L^−1^. Furthermore, *Enterococcus faecalis* and *Curtobacterium flaccumfaciens* pv. *flaccumfaciens* were the most sensitive food and plant pathogenic bacteria, respectively. Gas chromatography–mass spectrometry analysis was conducted to assign the ingredients present in the oil; 34 different components representing 95.71% of the total oil were identified, with n-hexadecanoic acid being the dominant component, followed by 2-Pentadecanone, 6,10,14-trimethyl, 1H-Indene, 1-ethylideneoctahydro-, and Cinnamyl tiglate. These findings demonstrate, for the first time, a broad spectrum of the antibacterial capacity of *E. billardieri*. Based on these observations, the oil could be applied as a natural preservative with the potential for designing novel products. Its bioactive agents can also be isolated for further use in the food and agricultural industries.

## Introduction

As the most taxonomically complex genus in the Apiaceae family (Calvino et al., 2008), *Eryngium* L. encompasses ~250 species growing worldwide, mainly in Asia, Europe, Australia, North Africa, and North and South America (Worz, 2004). Additionally, 10 species of this genus are distributed in Iran (Rezvani and Zaefarian, 2017; Sepanlou et al., 2019).

Various species of the genus *Eryngium* have been reported to possess ornamental, culinary, agricultural, and medicinal applications. The aerial parts of several members of this taxon are reportedly reliable sources of diverse secondary metabolites, such as polyacetylenes, flavonoids and saponins, coumarins, and monoterpene glycosides (Cárdenas-Valdovinos et al., 2023).

*Eryngium billardieri* Delar is a herbaceous and perennial species of the genus *Eryngium* (Pérez-Muñoz et al., 2022). It has been progressively elucidated that *E. billardieri* is a palliative, particularly for arthritis pain (Sharififar et al., 2014), as well as a natural antifungal bioagent (Abbasi et al., 2012). In addition to these purported traditional uses, the plant is recommended as a remedy for constipation (Mosaddegh et al., 2012) and acts as a potential antidiabetic agent as well (Khani et al., 2021).

It is quite obvious that phytopathogens along with foodborne bacteria significantly contribute to the overall loss in crop yields worldwide (Mansfield et al., 2012; Hameed et al., 2018). Sustainable food production through biological methods is an inexpensive substitute for conventional physical and non-biodegradable chemical methods (Allafchian et al., 2022; Soltani Nejad et al., 2023), which aims to hinder the development of a multiple resistance crisis among pathogenic microorganisms in relation to these substances (Karimi et al., 2019), preventing their extended environmental pollution and reduced effectiveness (Giaouris et al., 2014).

Contaminated food products, primarily caused by microbiological contamination, are responsible for more than 600 million disease incidences and 420,000 deaths each year (WHO, 2020). Additionally, plants are constantly threatened by a variety of pathogenic microorganisms that are present in their environments. The increasing social and economic implications of bacterial phytopathogens mean that there is a pressing need to produce safer foods and develop novel plant-based antibacterial blends. In general, plant-derived essential oils (EOs) have gained momentum for research purposes as non-phytotoxic compounds are potentially effective against plant pathogenic bacteria (Bajpai et al., 2011). Being comparatively harmless, these compounds have motivated the focus of innovative researchers for treating distinct challenging diseases (Hajian-Maleki et al., 2019; Allafchian et al., 2022), and several researchers have extensively documented the antimicrobial properties of EOs and their constituents (Nazzaro et al., 2013; Chouhan et al., 2017; Raveau et al., 2020).

Many plant-derived EOs can act as herbicides, fungicides, and bactericides and, therefore, could be beneficial for their management (Dayan et al., 2009; Mohd Israfi et al., 2022). These biomaterials can either inhibit microbial growth or cause their mortality with minimal toxicity to host cells, which ultimately leads to eliminating food contamination and waste (Karimi et al., 2019).

Existing phytochemical investigations indicate the presence of EOs in several *Eryngium* species, including *E. billardieri* (Sefidkon et al., 2004). However, to the best of our knowledge, the oil of this species has not been investigated for its potential content of antibacterial entities. Nonetheless, in some studies, volatile oils and botanical extracts of some other members of the genus *Eryngium* have been found to be effective sources against bacteria and fungi detrimental to food systems (Thiem et al., 2010; Dehghanzadeh et al., 2014; Merghache et al., 2014; Landoulsi et al., 2016; Sadiq et al., 2016; Daneshzadeh et al., 2019; Medbouhi et al., 2019; Kikowska et al., 2020; Mirahmadi et al., 2020; Nejati et al., 2021). Moreover, there are few available scientific studies on the antibacterial capacity of the EO of *Eryngium caeruleum* against phytopathogenic bacteria (Dehghanzadeh et al., 2014).

The agro-food sector not only serves as the primary source of nutrition for livestock but also provides more than 80% of the food consumed by human beings. Thus, indubitably, the management of infection risks associated with agro-food products is essential to achieve food security (The World Bank, 2022). This study aims to accomplish a multi-perspective study to reveal, for the first time, the antibacterial potential of *E. billardieri* oil against a range of prevalent harmful agro-food bacteria by analyzing the detailed chemical profile of the oil.

## 2. Materials and methods

### 2.1. Bacterial strains, media, and cultivation conditions

The plant pathogenic and food contaminant bacteria used in the assays are listed in Table 1. The food strains were purchased from the Iranian Biological Resource Center, Tehran, Iran. The phytopathogenic strains were obtained from the collection of the Plant Pathology Department, Tarbiat Modares University, Tehran, Iran and the Iranian Research Institute of Plant Protection, Tehran, Iran.

**Table 1:** List of bacterial strains used in this study.

**Bacteria**	**Family**	**References**	**Gram stain**
**Food pathogens**			
*Bacillus cereus*	*Bacillaceae*	IBRC^a^	+
*Bacillus subtilis*	*Bacillaceae*	IBRC	+
*Enterococcus faecalis*	*Enterococcaceae*	IBRC	+
*Escherichia coli*	*Enterobacteriaceae*	IBRC-RQC^b^	–
*Klebsiella pneumoniae*	*Enterobacteriaceae*	IBRC	–
*Listeria monocytogenes*	*Listeriaceae*	IBRC	+
*Salmonella enterica*	*Enterobacteriaceae*	IBRC	–
*Salmonella typhi*	*Enterobacteriaceae*	IBRC	–
*Proteus mirabilis*	*Enterobacteriaceae*	IBRC	–
*Pseudomonas aeruginosa*	*Pseudomonadaceae*	IBRC	–
*Staphylococcus aureus*	*Staphylococcaceae*	IBRC	+
**Phytopathogens**			
*Brenneria goodwini*	*Enterobacteriaceae*	PPD, TMU	–
*Brenneria nigrifluens*	*Enterobacteriaceae*	Falahi Charkhabi et al., 2010	–
*Burkholderia gladioli*	*Burkholderiaceae*	IRIPP^d^	–
*Curtobacterium flaccumfaciens* pv. *flaccumfaciens*	*Microbacteriaceae*	Osdaghi et al., 2018	+
*Erwinia amylovora*	*Enterobacteriaceae*	PPD, TMU	–
*Pectobacterium carotovorum* subsp. *carotovorum*	*Enterobacteriaceae*	Baghaee-Ravari et al., 2011	–
*Pseudomonas amygdali*	*Pseudomonadaceae*	PPD, TMU	–
*Pseudomonas savastanoi*	*Pseudomonadaceae*	IRIPP	–
*Pseudomonas syringae*	*Pseudomonadaceae*	Mosivand et al., 2009	–
*Ralstonia solanacearum*	*Pseudomonadaceae*	PPD, TMU	–
*Rhizobium radiobacter*	*Rhizobiaceae*	PPD, TMU^c^	–
*Xanthomonas* sp.	*Xanthomonadaceae*	PPD, TMU	–
*Xanthomonas translucens* pv. *undulosa*	*Xanthomonadaceae*	Falahi Charkhabi et al., 2017	–

^a^Iranian Biological Resource Center, Tehran, Iran.

^b^Routine Quality Control bacterium recommended in antimicrobial susceptibility testing standards by CLSI.

^c^Plant Pathology Department, Tarbiat Modares University, Tehran, Iran.

^d^Iranian Research Institute of Plant Protection, Tehran, Iran.

Prior to each stage, the strains were revived by streaking them on lysogeny broth (LB) agar plates. Subsequently, a single colony of each bacterium was used to inoculate tubes containing sterile liquid LB; these tubes were then placed in a shaking incubator (Behdad, Labtron Co., Tehran, Iran) overnight at their corresponding temperatures, as recommended by the Clinical and Laboratory Standards Institute (CLSI, 2021). Additionally, glycerol stock cultures of the bacteria were prepared in luria broth and 40% glycerol solutions and stored at −80°C.

### 2.2. Plant material sampling and oil extraction

*E. billardieri* seeds (IBRC-800) were obtained from Acer Research Complex, Iranian Biological Resource Center, Tehran, Karaj. The seeds were planted under greenhouse conditions (Relative humidity 70–80%, temperature: 22 ± 2°C, and light: 16 h light and 8 h dark) at the Faculty of Agriculture, Tarbiat Modares University, Tehran, Iran (Vardavard region, Latitude: 51°9′52.29″N, Longitude: 35°44′28.72″E). The aerial parts of the plants were collected during the vegetative phase, air-dried in the shade for 10 days, and ground. Subsequently, the plant materials were subjected to hydrodistillation with 250 ml of sterile distilled water using a Clevenger's apparatus (consisting of a round-bottomed flask, a tube for oil accumulation, and a reflux condenser) for 4 h, based on the standard procedure of Mirahmadi et al. (2020). The oily layer obtained on top of the aqueous distillate was separated, and anhydrous sodium sulfate was used to remove water after extraction. Subsequently, the EO was filtered and stored in a dark airtight container at 4–6°C until its use for further bioassays. Tween 20 surfactants (1%; diluted in 20% Ethyl alcohol) were added to the volatile oil at a ratio of 1:1 to improve the oil's distribution within the liquid medium.

### 2.3. Chemical analysis of the EO

The EO constituents were identified and quantified by GC (gas chromatography) and GC–MS (gas chromatography–mass spectrometry) using an Agilent 5975 gas chromatograph (Agilent Technologies Inc., Santa Clara, United States), equipped with an HP-5 MS narrow bore column (30 m × 0.25 mm, 0.25 μm film thickness) interfaced with a quadruple mass detector and a Wiley 7n. l computer library. The oven temperature was programmed at 40°C for 5 min, 50–250°C at 3°C min^−1^, and afterward, it was held at 250°C for 10 min. Furthermore, 2.0 μL of the extracted oil sample diluted in acetone (1/100, vol/vol) was injected manually in the split mode (ratio 1:10) at 250°C, with helium carrier gas at a flow rate of 0.06 L h^−1^. The MS was operated in electronic ionization energy set at 70 eV, 150 μA ionization current, 280°C ion source temperature, and mass range 35–450 atomic mass units (AMU).

The qualification of the extracted oil components was performed by calculating their retention indices under temperature-programmed conditions for n-alkanes (C8–C20) and the oil on an HP-5 MS column. The resulting individual ingredients were identified by comparing their mass spectra and retention indices (RI) with those of authentic samples and data already available in NIST and Wiley mass spectral libraries. The relative amount of the individual constituents was quantified by employing the area percentage method without considering the calibration factor.

### 2.4. Disk diffusion assay

The agar disk diffusion method based on the methodology developed by CLSI (2021) was adopted with slight modifications for the primary estimation of the antibacterial potency of the EO. Concisely, sterile paper disks (6 mm diameter, Whatman paper No. 1, Oxoid) were loaded with 10 mg of the EO and applied to the surface of LB agar plates. Then, 100 μL of overnight growth bacterial inoculum suspension (standardized to yield a concentration of 1 × 10^8^ CFU mL^−1^) was spread over the surface of the plates using sterile cotton swabs to ensure uniform microbial growth. Negative control disks received the same amount of sterile distilled water and Tween 20 surfactant (1%; diluted in 20% ethyl alcohol), and a streptomycin antibiotic (10 μg per disk) was used as the positive control. The plates were then kept at optimum characterized temperatures (CLSI, 2021) for 18 h, followed by the measurement of the diameter of growth inhibition zones with a caliper (including the disk diameter) expressed in millimeters (mm). The assay was repeated twice, each with four replications.

### 2.5. Determination of the concentration effect

The concentration effect was studied to ascertain the doses of the test substance possessing bacteriostatic and bactericidal influence on the growth of the isolates. The assessments were accomplished by following the broth microwell dilution procedure, according to the model described by CLSI (2015); only a few modifications were implanted. The trial was performed in 96-well polystyrene microtiter trays with a final volume of 220 μL in each microplate well. In brief, 12 2-fold serial dilutions of the EO were prepared in sterile LB media, and 200 μL of each dilution was added to the consecutive wells of 96-well polystyrene microtiter dishes. Inocula of the microorganisms prepared from 12 h cultures of each strain were diluted and adjusted to approximately 1 × 10^8^ CFU mL^−1^, and 20 μL of each suspension was distributed into each well. Positive (bacterial suspension + LB) and negative controls (1% Tween 20 + LB) were also included in the trays. The plates were incubated for 20 h at the respective temperature suggested for each bacterium (CLSI, 2021) under agitation. The EO's minimum inhibitory concentration (MIC) for each strain was defined as the lowermost value with no visible growth detected after incubation.

Using the observations of the MIC bioassay, 10 μL of the contents of each well with no visible growth was plated onto plates containing a solid LB medium. The lowest EO value where no viable bacteria were monitored on the plates after 24 h of incubation was designated as the minimal bactericidal concentration (MBC). The MIC and MBC values were estimated in two independent experiments, each with four replicates for every treatment × bacteria combination.

### 2.6. Statistical analysis

Experimental results in the present study were expressed as means ± standard deviations of four replicate measurements. The SPSS statistical package (version 22.0, SPSS Inc., Chicago, IL, USA) was employed to process the data, and an analysis of variance was performed to assess the differences between the groups. A value of *P* ≤ 0.05 was considered significant. Means were compared by performing Duncan's multiple range test.

## 3. Results

### 3.1. Chemical composition of the EO

**Figure 1:**
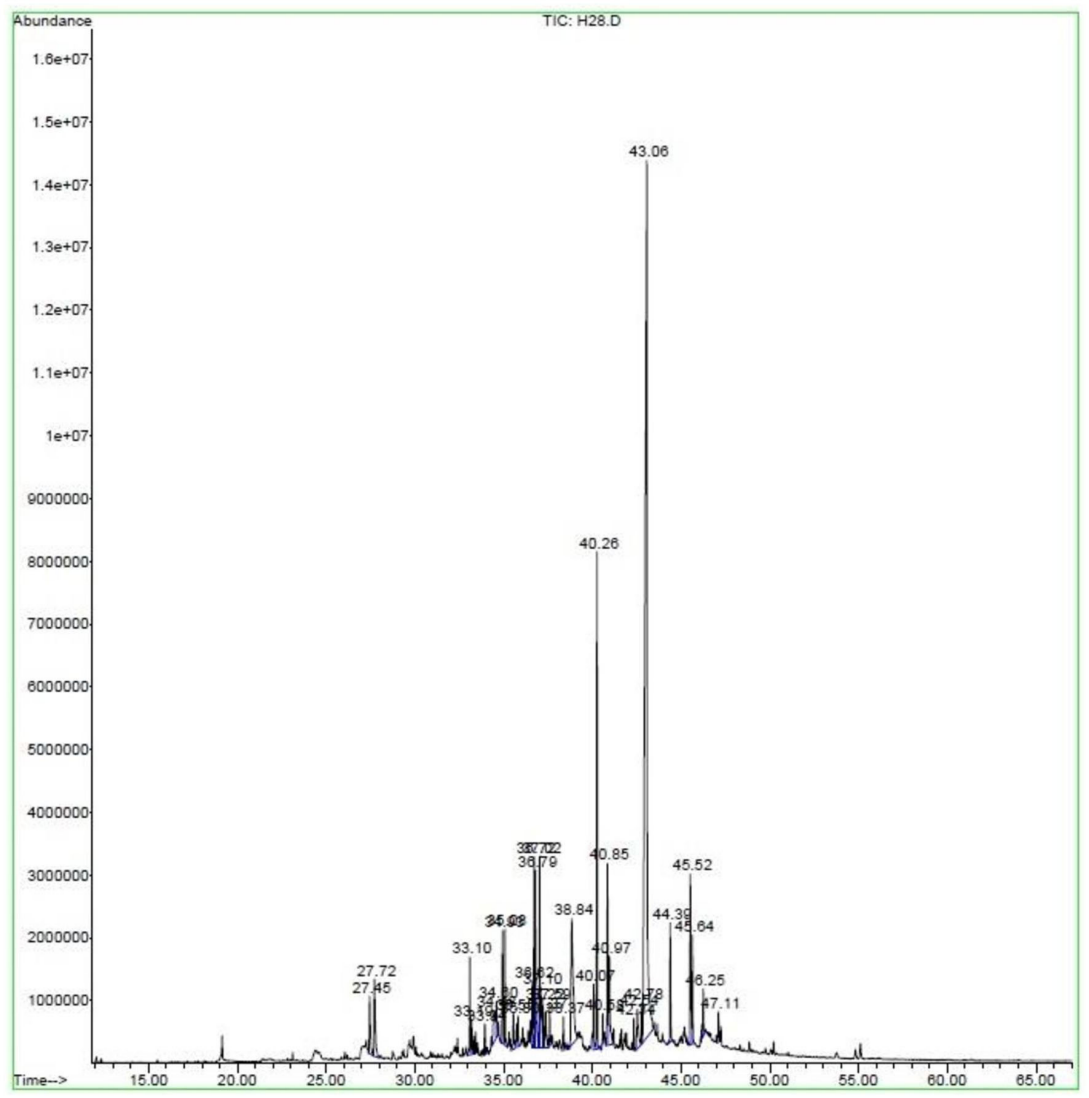
Gas chromatogram of *Eryngium billardieri* essential oil extracted by hydrodistillation.

The qualitative and quantitative chemical profiles of *E. billardieri* EO were analyzed by GC and GC–MS; the detailed volatile constituents, their retention time (RT), and the percentage of each component are given in Table 2 and shown graphically in Figure 1. Thirty-four different compounds were identified in the EO, representing 95.71% of the total oil. As shown in Figure 1, n-hexadecanoic acid is distinguished from the other components depicted in the chromatogram by a sharp peak (Retention time = 43.06), which indicates its high abundance in the extracted EO.

**Table 2:** GC–MS (gas chromatography–mass spectrometry) analysis results for *E. billardieri* chemical composition of the *E. billardieri* essential oil, as determined by GC–MS.

**Peak number**	**RT^a^**	**Compound**	**Area (%)**
1	27.45	Thymol	1.30
2	27.72	3-Methyl-4-isopropylphenol	2.12
3	33.10	syn-p-Fluorobenzaldehyde oxime	1.42
4	33.19	Methanone, dicyclohexyl-	0.58
5	33.94	Spiro[4.5]decan-6-one	0.54
6	34.47	Dodecanoic acid	2.19
7	34.93	(–)-Spathulenol	1.69
8	35.08	Caryophyllene oxide	1.68
9	35.55	Phosphite, di-(+)-menthyl-(–)-menthyl-	0.79
10	35.81	2,5-Octadecadienoic acid, methyl ester	0.53
11	36.62	2-Furanmethanol, tetrahydro-	1.15
**12**	**36.72**	**Cinnamyl tiglate**	**3.01**
**13**	**36.79**	**1H-Indene, 1-ethylideneoctahydro-**	**3.78**
14	37.02	Apiol	2.75
15	37.10	Caryophyllene oxide	1.54
16	37.23	Epiglobulol	1.02
17	37.37	Aristolene epoxide	0.87
18	37.59	6-Isopropenyl-4,8a-dimethyl-1,2,3,5,6,7,8,8a-octahydro-naphthalen-2-ol	0.67
19	38.37	Aromadendrene oxide-(1)	0.62
**20**	**38.84**	**Tetradecanoic acid**	**6.19**
21	40.07	Bicyclo[3.1.1]heptane, 2,6,6-trimethyl-	1.00
**22**	**40.26**	**2-Pentadecanone, 6,10,14-trimethyl**	**6.67**
23	40.58	1,13-Tetradecadiene	0.31
24	40.85	Phthalic acid, butyl isohexyl ester	2.38
25	40.97	Pentadecanoic acid	2.55
26	42.34	Sulfurous acid, octyl 2-pentyl ester	0.45
27	42.54	cis-9-Hexadecenal	1.36
28	42.78	Dibutyl phthalate	0.52
**29**	**43.06**	**n-hexadecanoic acid**	**38.81**
30	44.39	Naphthalene, 2-methyl-1-nitro-	1.78
31	45.52	Isophytol	2.73
32	45.64	1,3,2-Oxazaborolane, 2-butyl-	1.66
33	46.25	Bicyclo[2.2.1]heptane-2-carboxylate	0.75
34	47.11	3-Fluoro-4-methoxyphenylacetic acid	0.55
**Total identified**			**95.71**

^a^RT: The time taken for a solute to pass through a chromatography column.

The main components (compromising more than 3% of the oil profile) are demonstrated in boldface.

The amount of each component is quantified in Table 2; the most predominant ingredient found in the oil was n-hexadecanoic acid in a proportion of 38.81 %, followed by 2-Pentadecanone, 6,10,14-trimethyl (6.67 %), 2-Pentadecanone, 6,10,14-trimethyl (6.19 %), 1H-Indene, 1-ethylideneoctahydro- (3.78 %), and Cinnamyl tiglate (3.01%). The other identified components were minor, ranging from 0.31 to 2.75%, and all were obtained from NIST and Wiley mass spectral libraries (Table 2).

### 3.2. Antibacterial screening by the disk diffusion method

**Figure 2:**
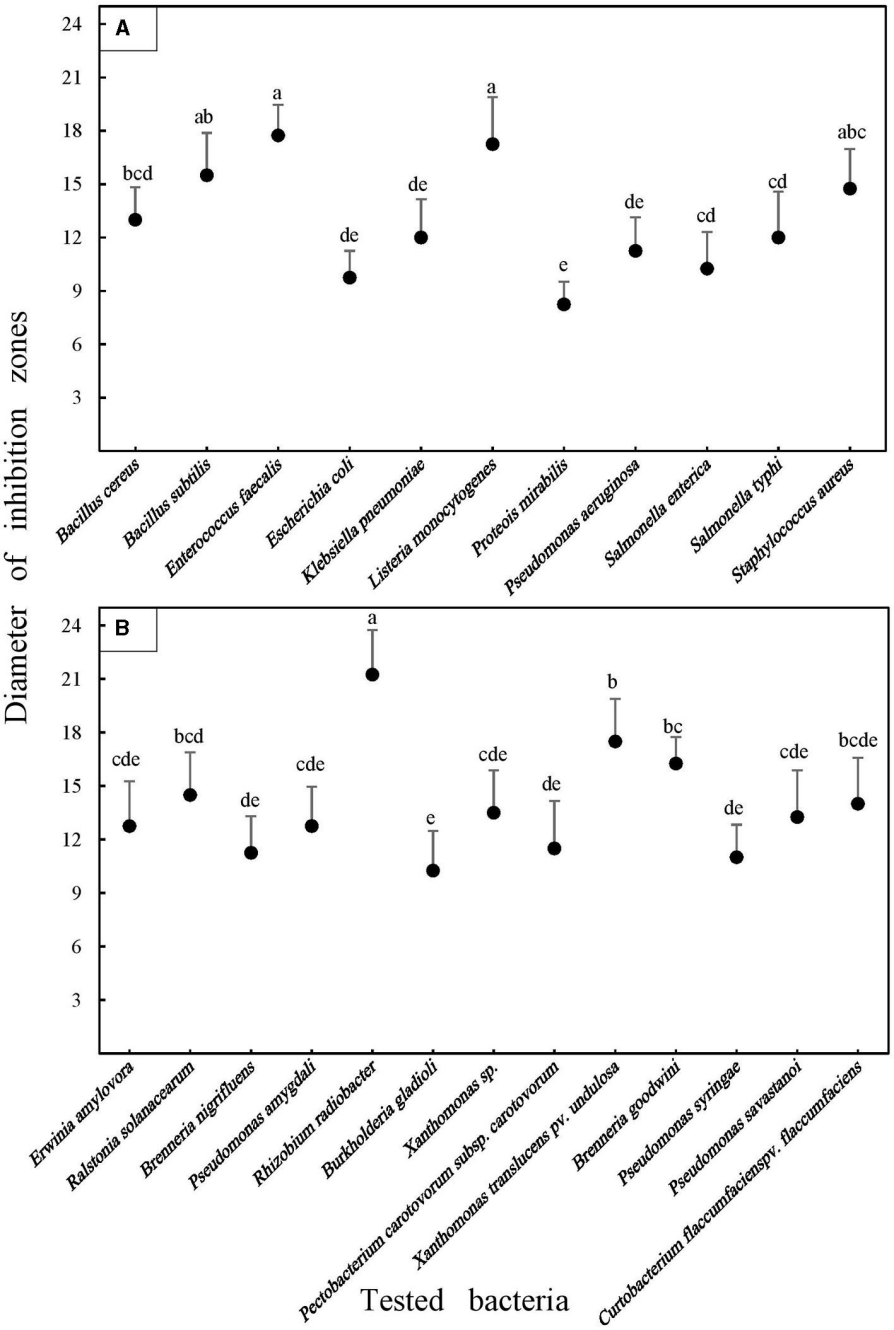
Average antibacterial activities of the *Eryngium billardieri* essential oil against food **(A)** and plant pathogenic **(B)** bacteria in the disk diffusion assay. Each disk contained 10 mg of the oil deposited in the center of Petri dishes containing solid LB media. Average values and standard deviations were obtained from at least four inoculations. Means were separated by performing Duncan's multiple range test. Different letters represent significant changes.

A preliminary estimation of the putative *in vitro* antibacterial potential of *E. billardieri* EO against the two different mentioned categories of harmful Gram-positive and Gram-negative bacteria (agro-foods) was conducted using an agar disk diffusion trial (Table 1). The results indicated in Figure 2 represent the inhibition area (mm) around the blank disks loaded with 10 mg of bioactive EO, which includes the diameter of the disk (6 mm). The appearance of transparent circular zones around disks after overnight incubation of Petri dishes was attributed to the lack of bacterial growth.

*Escherichia coli*, a well-known bacterial species in the Enterobacteriaceae family, was used as the quality control strain following CLSI recommendations in antibacterial bioassays. Interestingly, however, this bacterium clearly responded to the adjustment of the EO, which was verified by the formation of a halo of ~9.75 mm around the disks placed at the center of the plates seeded by this pathogen. As depicted in Figure 2, the highest abundance of the inhibition diameter valuations was observed within the range of 11–13 mm. Nine different analyzed pathogens were within this range, including five phytopathogens, namely, *Rhizobium radiobacter* (12.75 mm), *Ralstonia solanacearum* (11 mm), *Brenneria nigrifluens* (11.25 mm), *Burkholderia gladioli* (12.75 mm), and *Pseudomonas amygdali* (11.5 mm). Additionally, four pathogens belonged to the food category, including *Bacillus cereus* (13 mm), *Pseudomonas aeruginosa* (11.25 mm), *Salmonella enterica* (12 mm), and *Salmonella typhi* (12 mm).

**Figure 3:**
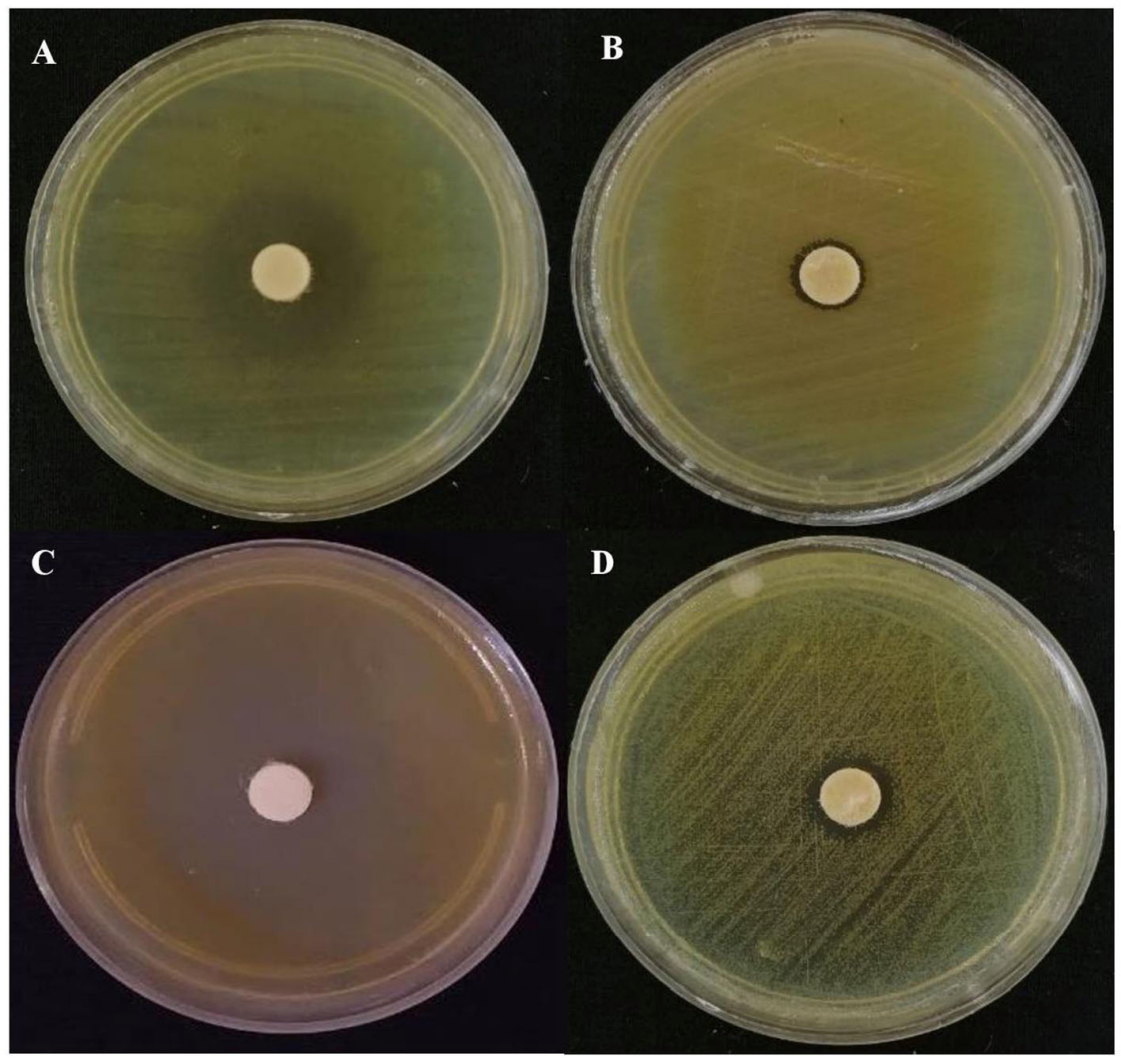
Representative images of the antibacterial activity of *Eryngium billardieri* EO against the most resistant and the most susceptible food pathogens [**(A)** *Enterococcus faecalis* and **(B)** *Proteous mirabilis*] and phytopathogens [**(C)** *Curtobacterium flaccumfaciens* pv. *flaccumfaciens* and **(D)** *Erwinia amylovora*] in the disk assay.

Interestingly, a broad variation in the magnitude of antibacterial properties of the EO was detected against exanimated agro-food contaminants (Figure 2). The range of the inhibition halo diameter varied from 8.25 mm (*Proteus mirabilis*) to 17.75 mm (*Enterococcus faecalis*) among harmful foodborne pathogens. In the case of phytopathogenic bacteria, the strongest activity was recorded in Petri dishes seeded by the Gram-positive phytopathogenic bacterium, *Curtobacterium flaccumfaciens* pv. *flaccumfaciens*, which was ~21.25 mm. However, the smallest diameter of the inhibition halo was detected in the plates cultured using *Erwinia amylovora*, with an average of 10.5 mm (Figure 3). Meanwhile, a weak inhibition (between ~1 and 3 mm) was observed in the negative control plates treated with 10 mg Tween 20 surfactant (1%; diluted in 20% ethyl alcohol).

### 3.3. Estimation of bactericidal and bacteriostatic values by the broth microdilution method

In the next stage, the bacteriostatic and bactericidal potential of the EO was evaluated and recorded quantitatively as MIC and MBC in Table 3. Among the assessed food contaminant bacteria, the strongest activity was exerted against *E. faecalis*, with the MIC recorded as 1.88 ± 0.51 g L^−1^. The least susceptible bacterium was *P. mirabilis* and it had an MIC equal to 34.17 ± 4.91 g L^−1^, ~18 times higher than that of *E. faecalis*, making it the most resistant isolate among all exanimated bacteria in the two different categories.

**Table 3:** Mean antibacterial activity of *E. billardieri* essential oil (g L^−1^) estimated in 96-well plates containing different values of the oil in a liquid LB medium.

**Bacterial strains**	**MIC**	**MBC**
*B. cereus*.	12.5 ± 4.18^d^	13.33 ± 4.08^d^
*B. gladioli*	25.83 ± 3.76^b^	25.83 ± 3.76^b^
*B. goodwini*	5.42 ± 1.02^e^	5.42 ± 1.02^e^
*B. nigrifluens*	27.5 ± 4.18^b^	27.5 ± 4.18^b^
*B. subtilis*	9.17 ± 3.76^de^	9.17 ± 3.76^de^
*C. f*. pv. *flaccumfaciens*	0.67 ± 0.23^f^	0.73 ± 0.23^f^
*E. faecalis*	1.88 ± 0.51^ef^	1.88± 0.51^ef^
*E. amylovora*	31.67 ± 5.16^a^	31.67 ± 5.16^a^
*E. coli*	28.33 ± 5.16^b^	28.33 ± 5.16^b^
*K. pneumoniae*	20.83 ± 3.76^c^	20.83 ± 3.76^c^
*L. monocytogenes*	2.29 ± 0.51^ef^	2.29 ± 0.51^ef^
*P. c*. subsp. *carotovorum*	15.83 ± 3.76^d^	15/.83 ± 3.76^d^
*P. mirabilis*	34.17 ± 4.91^a^	35 ± 4.47^a^
*P. aeruginosa*	24/17 ± 3.43^c^	24.17 ± 3.43^c^
*P. amygdali*	21.67 ± 4.08^c^	23.33 ± 2.58^c^
*P. savastanoi*	0.88 ± 0.24^f^	0.93 ± 0.34^f^
*P. syringae*	3.33 ± 0.64^ef^	3.33 ± 0.64^ef^
*R. solanacearum*	29.17 ± 3.76^ab^	29.17 ± 3.76^ab^
*R. radiobacter*	20 ± 3.16^c^	20.83 ± 2.04^c^
*S. enterica*	32.5 ± 4.18^a^	32.5 ± 4.18^a^
*S. typhi*	23.33 ± 4.08^c^	24.17 ± 4.91^c^
*S. aureus*	21.67 ± 5.16^c^	21.67 ± 5.16^c^
*Xanthomonas* sp.	15 ± 3.16^d^	15 ± 3.16^d^
*X. t*. pv. *undulosa*	12.5 ± 4.18^d^	14.17 ± 3.76^d^

MIC, minimum inhibitory concentration, g L^−1^; MBC, minimum bactericidal concentration, g L^−1^.

The trial was repeated twice, and the average and standard error of at least four measurements were reported. Means were separated by performing Duncan's multiple range test. Different letters indicate significant changes.

The frequency of MIC values was higher, between 20 and 30 g L^−1^ (*Klebsiella pneumoniae*: 20.83 ± 3.76 g L^−1^, *P. mirabilis*: 28.33 ± 5.16 g L^−1^, *P. aeruginosa*: 24.17 ± 3.43 g L^−1^, *S. typhi*: 23.33 ± 4.08 g L^−1^, and *Staphylococcus aureus*: 21.67 ± 5.16 g L^−1^). In most cases, MBCs were equal to MICs, except for *B. cereus* (MIC: 12.5 ± 4.18 and MBC: 13.33 ± 4.08), *P. mirabilis* (MIC: 34.17 ± 4.91 and MBC: 35 ± 4.47), and *S. typhi* (MIC: 23.33 ± 4.08 and MBC: 24.17 ± 4.91), in which the required lethal concentrations were slightly higher than the bacteriostatic values (Table 3).

Similarly, the EO of *E. billardieri* showed remarkable antibacterial capability on the tested agricultural microorganisms. Relatively lower inhibitory and lethal concentrations (lower than 5 g L^−1^) compared to the other strains were screened against *Pseudomonas syringae, Pseudomonas savastanoi*, and *C. f*. pv. *flaccumfaciens*, which were recorded as 3.33 ± 0.64 5 g L^−1^, 0.88 ± 0.24 g L^−1^, and 0.67 ± 0.23 g L^−1^, respectively. *E. amylovora* could be considered the most resistant bacterium in this group, with its MIC and MBC values similar to each other and equal to 31.67 ± 5.16 g L^−1^. Among other analyzed agricultural pathogens, the MICs and MBCs ranged from 12.5 ± 4.18 g L^−1^ to 29.17 ± 3.76 g L^−1^ and 14.17 ± 3.76 g L^−1^ to 29.17 ± 3.76 g L^−1^, separately. The latter was assigned to *R. solanacearum*, while the former was related to *Xanthomonas translucens* pv. *undulosa*. It was observed that the MICs for this pathovar, along with *P. savastanoi, P. amygdali, C. f*. pv. *flaccumfaciens*, and *R. radiobacter*, were slightly lower than the MBCs (Table 3).

## 4. Discussion

Over the past decades, agricultural products (such as vegetables and fruits) have been reported as the major carriers of foodborne diseases, the main agents limiting food resources and threatening food security. The spread of infectious diseases due to foodborne pathogens poses a global threat to human health and the economy. Exposure of agro-food crops to distinct pathogens from “farm to fork” can lead to their contamination, which could increase the worldwide burden of diseases on account of the fact that food crops act as a link between infectious agents and humans upon acquisition (Fones et al., 2020; Brunn et al., 2022; Nehra et al., 2022). To overcome these challenges, botanical products such as EOs have been proposed as a reliable source of bioactive organic ingredients. Recently, there has been a renewed interest in investigating the efficiency of natural agents, which is a relatively new but rapidly growing approach (Shaheen and Issa, 2020). This study can be considered the first concerning the antibacterial capacity of the EO of *E. billardieri*, and its observations were promising. Our outcomes illustrated that the pale-green EO of this herb can be introduced as a novel source of antimicrobial substances with approximately moderate to high efficiency. Both agricultural and food pathogens were controlled when this biomaterial was applied in solid and liquid media, although the degree of sensitivity varied.

The effectiveness of crude EO is associated with the presence of multiple diverse antimicrobial compounds, with a typical oil containing 20–60 bioactive ingredients. This results in the effectiveness of the specificity of each EO against various microorganisms (Swamy et al., 2016).

The GC–MS analysis revealed that n-hexadecanoic acid was the principal component of the analyzed bioactive oil and is probably responsible for the inhibitory effects observed. According to Sefidkon et al. (2004), alpha-muurolene and beta-gurjunene are the most abundant constituents of *E. billardieri* EO; moreover, the authors detected alpha-copaene, beta-elemene, beta-selinene, valencene, delta and beta-cadinene, spathulenol, globulol, virdiflorol, and alpha-eudesmol in appreciable amounts, whereas we detected only spathulenol and globulol from these compounds in different proportions. In another study, octanoic acid, clovanediol, epiglobulol, isoxazole, carvone, and thymol were reported to be the major compounds of this oil; nevertheless, only epiglobulol and thymol were found to be common with our own data (Chamgordani et al., 2023). It is often quite challenging to compare the results obtained from different studies because the compositions of the essential oils could vary greatly depending on the consequences of several factors, such as geographical location, seasons, climate conditions, growth regulators, presence of different chemotypes, individual genetic variations, plant age, developmental stages, diversity of plant parts, harvesting periods, and the methods used for drying and extracting the oils (El Abed et al., 2014; Hajian-Maleki et al., 2021).

Based on the size of inhibition zones observed in the disk assay, the antimicrobial capacity of an EO could be divided into three classes (Rota et al., 2008): weak potential (inhibition zone ≤ 12 mm), moderate potential (12 mm < inhibition zone <20 mm), and strong potential (inhibition zone ≥ 20 mm). Based on this division, in the disk diffusion assay performed in this study, the *E. billardieri* EO exerted strong activity against the Gram-positive bacterium *C. f*. pv. *flaccumfaciens*, which was the only agricultural species analyzed. A moderate effect was exerted against *B. cereus, Bacillus subtilis, E. faecalis, Listeria monocytogenes, S. aureus, R. radiobacter, B. goodwini, B. gladioli, Pectobacterium carotovorum* subsp. *carotovorum, P. savastanoi, B. goodwini, P. syringae, Xanthomonas* sp., and *X. t*. pv. *undulosa*. Finally, it could be inferred that the *E. billardieri* EO acted weakly against the remaining strains, namely, *E. coli, K. pneumoniae, P. mirabilis, P. aeruginosa, S. enterica, S. typhi, B. nigrifluens, E. amylovora, P. amygdali*, and *R. solanacearum*. Upon initial observation, similar evidence had been obtained in previous studies regarding the antibacterial potential of EOs and extracts from various species of *E. billardieri* against different bacteria. In the case of herbal extracts, it has been reported that ethanolic extracts of leaves and roots of three species of the *Eryngium* genus, namely, *Eryngium planum, Eryngium campestre*, and *Eryngium maritimum*, can inhibit the growth of *S. aureus* to a greater degree than *B. subtilis* (Thiem et al., 2010). Methanolic extract of *E. caeruleum* was reported to be active against a prominent antibacterial activity against six bacterial strains, including *E. faecalis, P. mirabilis, E. coli, S. typhi, K. pneumoniae*, and *P. aeruginosa* (Sadiq et al., 2016).

Regarding monitoring the antibacterial potential of the EO of *Eryngium* species, *E. campestre, Eryngium amethystinum*, and *Eryngium palmatum* oils were found to be active against a number of foodborne bacteria, expressing the highest potential against Gram-positive bacteria *S. aureus* and Gram-negative *K. pneumoniae* and *P. mirabilis* (Matejić et al., 2018). In this context, Mirahmadi et al. (2020) investigated the antimicrobial capacity of *E. caeruleum* EO against five foodborne pathogenic bacteria and showed that *S. aureus* was the most sensitive while *E. coli* was the most resistant among these bacteria. In a recent study, it was demonstrated that *E. campestre* EO could exert a strong influence against Gram-positive strains, such as *S. aureus, B. cereus*, and *E. faecalis* (Medbouhi et al., 2019).

Intriguingly, the inhibition diameters in the disk assay accorded with the bacteriostatic and bactericidal assessments. In other words, broader inhibition areas correlated with lower needed MICs and MBCs in most cases. However, there were some exceptions to this trend. The pattern recognized in this study was similar to the findings of Bassolé et al. (2011). MICs and MBCs were frequently identical, although in some cases, MBCs were higher than their corresponding MICs. In these cases, it seems feasible that the EO applied inhibition against the bacteria via killing activity, which is consistent with the findings of Kim et al. (1995).

The precise mechanism of action of several EO constituents is still not known, although pioneering studies in the past have provided some insights. The discrepancy in the susceptibility of microorganisms to the EO could be ascribed to their action mode, variations in the target site, such as cell membrane vs. cell wall, or their rate of penetration through the target site (Dorman and Deans, 2000). Factors determining the level of EO potency are composition, lipophilic properties, low or high aqueous solubility, functional groups present in active components, and their synergistic effects (Cox et al., 2000; Chouhan et al., 2017).

Not as a permanent pattern, but it could be construed that a more vigorous influence was imposed on Gram-positive species compared to Gram-negative species. It could be deduced from the results of both antibacterial trials that Gram-positive tested isolates, namely, *B. cereus, B. subtilis, C. f*. pv. *flaccumfaciens, E. faecalis, L. monocytogenes*, and *S. aureus*, were more significantly inhibited by the application of the EO. It has been well-documented that Gram-positive bacteria are more susceptible to EOs than Gram-negative bacteria (Huang et al., 2014), which aligns with our findings. This could be attributed to the fact that Gram-negative bacteria have a rigid, more complicated outer cell membrane that is rich in lipopolysaccharide (LPS), restricting the flow rate of hydrophobic EOs into the intracellular environment, while this extremely complex structure is absent in Gram-positive bacteria. In contrast, Gram-positive bacteria are surrounded by a thick peptidoglycan wall that is not dense enough to resist small antimicrobial molecules, facilitating accessibility to the membrane. Moreover, the infiltration of hydrophobic ingredients of the EOs is facilitated in Gram-positive bacteria due to the lipophilic ends of lipoteichoic acid present in their cell membranes (Joshi et al., 2015; Maurya et al., 2021).

## 5. Conclusion

In conclusion, our preliminary evidence confirmed the antimicrobial activity of *E. billardieri* EO, justifying its potential use against bacterial contaminants. This opens up the possibility of its application in agricultural systems and the food chain for generating biocidal compositions by minimizing the risks of traditional sanitizers and synthetic antimicrobial materials. In this way, environmental risks would be significantly lower due to the volatile nature of EOs, resulting in decreased persistency compared to synthetic pesticides. Ultimately, further research is necessary to develop a viable commercial methodology, taking into account environmental, health, and safety considerations, as well as associated hazards and risks.

## Data availability statement

The original contributions presented in the study are included in the article/supplementary material, further inquiries can be directed to the corresponding author.

## Author contributions

HH-M carried out all experiments and wrote the manuscript. MS-b supervised the project and edited the manuscript. All authors contributed to the article and approved the submitted version.
